# Pharmacokinetic Comparisons of Benzoylmesaconine in Rats Using Ultra-Performance Liquid Chromatography-Tandem Mass Spectrometry after Administration of Pure Benzoylmesaconine and Wutou Decoction

**DOI:** 10.3390/molecules191016757

**Published:** 2014-10-17

**Authors:** Pei-Min Dai, Ying Wang, Ling Ye, Shan Zeng, Zhi-Jie Zheng, Qiang Li, Lin-Liu Lu, Zhong-Qiu Liu

**Affiliations:** 1 Department of Pharmaceutics, School of Pharmaceutical Sciences, Southern Medical University, Guangzhou 510515, Guangdong, China; E-Mails: daipeimin1989@163.com (P.-M.D.); IITCM_Wangy@126.com (Y.W.); lotus623@126.com (L.Y.); zengshan_jessica@126.com (S.Z.); zhijiezheng@126.com (Z.-J.Z.); liqiangsmu@163.com (Q.L.); iitcm_lu@126.com (L.-L.L.); 2 International Institute for Translational Chinese Medicine, Guangzhou University of Chinese Medicine, Guangzhou 510006, Guangdong, China

**Keywords:** benzoylmesaconine, Wutou decoction, pharmacokinetics, UPLC-MS/MS

## Abstract

Wutou decoction is widely used in China because of its therapeutic effect on rheumatoid arthritis. Benzoylmesaconine (BMA), the most abundant component of Wutou decoction, was used as the marker compound for the pharmacokinetic study of Wutou decoction. The aim of the present study was to compare the pharmacokinetics of BMA in rats after oral administration of pure BMA and Wutou decoction. Pure BMA (5 mg/kg) and Wutou decoction (0.54 g/kg, equivalent to 5 mg/kg BMA) were orally administered to rats with blood samples collected over 10 h. Quantiﬁcation of BMA in rat plasma was achieved using sensitive and validated ultra-performance liquid chromatography-tandem mass spectrometry (UPLC-MS/MS). Specifically, the half-life (*T*_1/2_) and mean residence time values of pure BMA were 228.3 ± 117.0 min and 155.0 ± 33.2 min, respectively, whereas those of BMA in Wutou decoction were decreased to 61.8 ± 35.1 min and 55.8 ± 16.4 min, respectively. The area under the curve (AUC) of BMA after administration of Wutou decoction was significantly decreased (five-fold) compared with that of pure BMA. The results indicate that the elimination of BMA in rats after the administration of Wutou decoction was significantly faster compared with that of pure BMA.

## 1. Introduction

Herbal medicine is mostly administered in combination based on the principle of traditional Chinese Medicine to achieve effect optimization or toxicity reduction. It is generally recognized that the combined use of prescriptions have a greater impact on the therapeutic effects. Pharmacokinetic study, a useful method to predict the* in vivo* process, has been used to elucidate the different processes between pure chemicals and multiple-ingredient prescriptions; therefore, it is important to conduct pharmacokinetic studies to evaluate the rationality of the traditional prescriptions.

Wutou decoction, a well-known traditional prescription, comprises Radix *Aconiti* (derived from the dried root of *Aconitum carmichaeli* Debx*.*), Herba *Ephedrae* (derived fromthe stem of *Ephedra sinica* Stapf*.*), Radix *Paeoniae Alba* (derived from the dried root of *Paeonia lactiflora* Pall.), Radix *Astragali* (derived from the dried root of *Astragalus membranaceus*) and Radix *Glycyrrhizae* (derived from the dried root of *Glycyrrhiza uralensis* Fisch). The decoction has been used for hundreds of years in China for rheumatoid arthritis (RA) treatment.

To date, many studies have investigated the effects of Wutou decoction in patients with RA. Radix *Aconiti*, a naturally occurring product, is considered a prominent component of Wutou decoction because of its therapeutic effects against RA [1,2]. However, Radix *Aconiti* is a toxic herb that can cause serious cardiac poisoning [3,4,5,6]. Previous studies revealed a 58% incidence rate of toxic reactions among 188 patients who had taken aconitum [7,8,9]. Aconitum alkaloids are the effective chemicals of Radix *Aconiti*. Notably, the high levels of toxicity of Radix *Aconiti* are derived from diester aconitum alkaloids, including aconitine, mesaconitine (MA) and hypaconitine, which can be hydrolyzed to benzoylaconine, benzoylmesaconine (BMA) and benzoylhypaconine, respectively. The MA hydrolysate BMA demonstrates lower toxicity [7], higher abundance and a better pharmacological effect than the other two monoester diterpenoid alkaloids in Wutou decoction [10]. This study selected BMA as a marker compound for the pharmacokinetic study of Wutou decoction.

BMA exhibits biological effects, including analgesic [11,12], antiviral and antifungal activities [13]. It can also stimulate cytokine secretion [14]. Although BMA is less toxic than diester diterpene alkaloids [10], excessive intake of BMA still causes toxic reactions [15]. Hence, several pharmacokinetic studies have been performed to understand the* in vivo* process of BMA by using the LC-MS method [16,17,18,19,20]. For instance, some researchers [16] found that BMA demonstrates fast absorption and elimination* in vivo* after the administration of pure BMA (*T*_1/2 β_ = 407 ± 180 min). Others [17] verified that BMA has short *T*_max_ (36.17 ± 1.72 min) compared with the other two monoester aconitum alkaloids after intravenous drop infusion of “SHEN-FU” injectable powder. Recently, research on the pharmacokinetics of BMA was conducted in combination with other herbs or components, such as Dahuang-Fuzi decoction and Radix *et Rhizoma Rhei* [18,19]. The pharmacokinetic parameters (C_max_, AUC) of BMA were remarkably reduced; *T*_1/2_ and mean residence time (MRT) were delayed with oral administration of Dahuang-Fuzi decoction [18]. The additional studies demonstrated that co-administration with Rhizoma *Zingiberis* could reduce the C_max_ of BMA with the increased *T*_1/2_, *T*_max_ and AUC [19]. The co-administration suggests that Rhizoma *Zingiberis* or other ingredients in Dahuang-Fuzi decoction could not accelerate the elimination of BMA, therefore having little influence on the toxicity reduction.

Although the above studies focused on the pharmacokinetics of BMA [16,17,18,19,20], little is known about the pharmacokinetics after the administration of Wutou decoction and pure BMA. Since Wutou decoction has complex ingredients, herb-herb interactions may exert an influence on the efficacy of Chinese medicine; therefore, this study aims to compare the pharmacokinetics of BMA after oral administration of pure BMA and Wutou decoction to rats using a rapid, sensitive and reliable UPLC-MS/MS method. The information obtained might be useful for understanding different herb-herb interactions between BMA and other ingredients, which can be used as a reference for the clinical administration of Wutou decoction.

## 2. Results and Discussion

### 2.1. Validation of UPLC-MS/MS Methods

**Figure 1:**
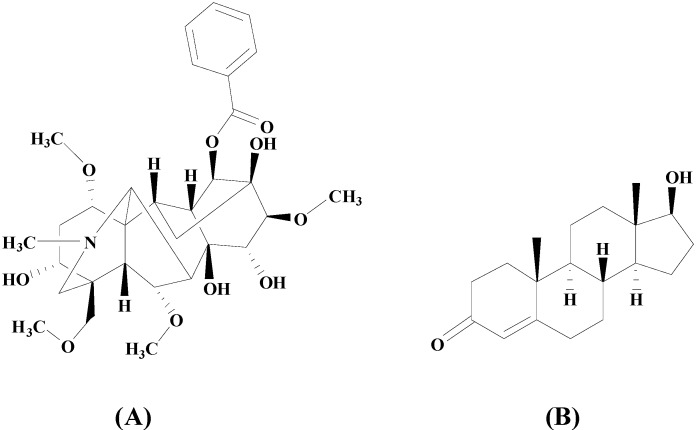
Chemical structures of benzoylmesaconine (BMA) (**A**) and the internal standard, testosterone (**B**).

An Acquity UPLC BEH C18 column was selected for the chromatographic separation of BMA (Figure 1A) and testosterone (Figure 1B), because of its good peak shapes and acceptable retention times. A 2-mM ammonium acetate and acetonitrile solution led to better peak shapes compared to other mobile phases. Testosterone was selected as an internal standard, because of its similar chromatographic behavior and complete separation with BMA.

The protein precipitation method developed in this study for the preparation of plasma samples was more simple and cost-effective compared with those developed in previous studies [17,21,22]. Several organic solvents, such as methanol, acetonitrile and ethyl acetate, were investigated for extraction. The results showed that BMA and testosterone could not be completely extracted simultaneously by acetonitrile, ethyl acetate and ethanol. Methanol was finally selected for sample preparation, because of its relatively high extraction recovery. Methanol-water (1:1), instead of the mobile phase, has been used to reconstitute the residue, because of the better peak shape. Therefore, a one-step process of protein precipitation with methanol that exhibited acceptable recovery was selected. This process was time saving and helpful to the stability of plasma samples.

**Figure 2:**
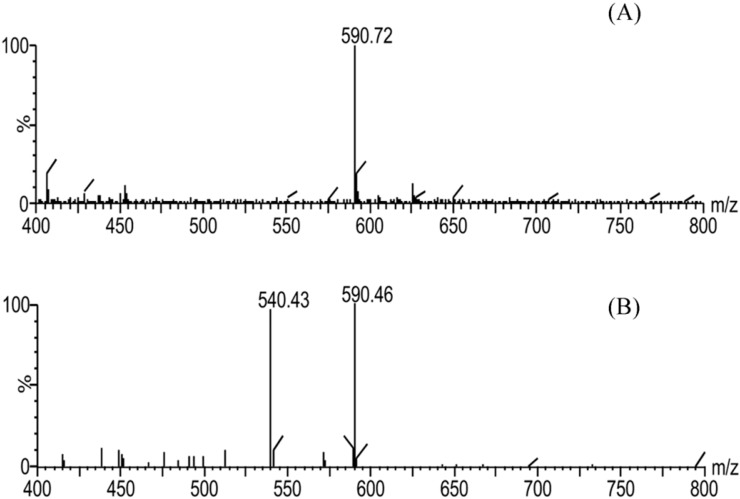
Full-scan (**A**) and MS/MS daughter scan (**B**) spectrograms of BMA.

**Figure 3:**
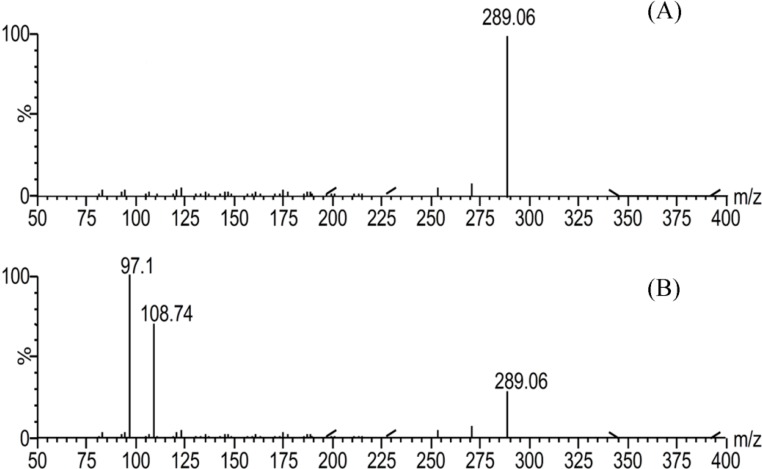
Full-scan (**A**) and MS/MS daughter scan (**B**) spectrograms of testosterone.

The concentrations of BMA in the plasma samples were too low to be determined by UPLC-UV. Thus, an UPLC-MS/MS method with higher sensitivity than UPLC-UV was developed in this study. The MS and MS/MS daughter scan spectrograms of BMA and testosterone in the ESI source are shown in Figure 2 and Figure 3, respectively.

**Figure 4:**
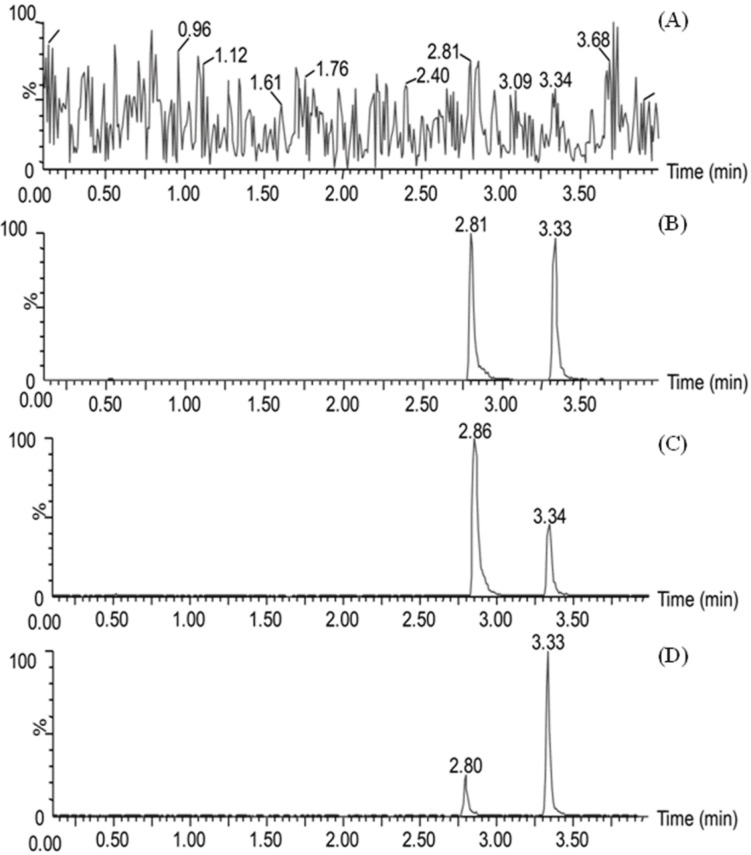
Chromatograms for BMA and testosterone in blank plasma (**A**); blank plasma spiked with BMA at 6 ng/mL and testosterone at 100 nM (**B**); plasma sample of BMA and testosterone after administration of pure BMA (**C**); and Wutou decoction (**D**). The retention time of BMA and testosterone was 2.8 and 3.3 min, which could be separated completely.

The chromatograms showed excellent peak shapes for BMA and testosterone at retention times of 2.8 and 3.6 min, respectively, with no endogenous interference. The peak responses of blank plasma samples spiked with BMA and testosterone are shown in Figure 4.

The calibration curve was investigated in the range of 0.3 ng/mL to 60 ng/mL. All curves had correlation coefficients of >0.990. The lower limit of quantification (LLOQ) of BMA under the developed UPLC-MS/MS method was 0.3 ng/mL, with a signal-to-noise ratio of >10.

The precision and accuracy of the method for detecting BMA were evaluated at three concentrations. The RSD% values for the intra-day and inter-day precision were smaller than 6.1% and 13.9%, respectively. The RE% values for the lowest concentration were 17.6% and smaller than 15.0% for the other two concentrations (see in Table 1).

**Table 1:** Accuracy and precision of BMA in rat plasma (n = 5).

Concentrations (ng/mL)	Accuracy (RE%)	Precision (RSD%)	
		Intra-day	Inter-day
1.2	17.6	6.1	13.9
6	12.7	6.1	7.9
30	10.3	3.7	4.7

The extraction recovery values of BMA were >85% at three concentrations. The matrix effects were over the range of 126.1% to 139.0% at low, middle and high concentrations, respectively, suggesting that significant ion suppression or enhancement did not occur at the expected retention times of the targeted ions (see in Table 2).

**Table 2:** Extraction recovery and matrix effect of BMA in rat plasma (n = 5).

Concentrations (ng/mL)	Extraction Recovery		Matrix Effect	
	Mean (%)	RSD (%)	Mean (%)	RSD (%)
1.2	92.0	5.4	126.1	5.9
6	92.9	9.3	139.0	2.3
30	85.2	6.4	134.1	6.5

The accuracy biases of BMA in the rat plasma samples ranged from 94.7% to 109.8%, 99.2% to 114.6%, 90.2% to 101.7% and 92.6% to 100.0% for the stability evaluation of short-term storage, long-term storage, three freeze-thaw cycles and auto-sampler storage, respectively (see in Table 3).

**Table 3:** Stability evaluation of BMA in rat plasma (n = 5).

Concentrations (ng/mL)	Short-Term Storage		Long-Term Storage		Three Freeze-Thaw Cycles		Auto-Sampler Stability	
	Accuracy (%)	RSD%	Accuracy (%)	RSD%	Accuracy (%)	RSD%	Accuracy (%)	RSD%
1.2	94.7	13.3	114.6	2.4	90.2	10.2	100.0	3.9
6	109.8	5.1	110.3	3.3	100.8	4.0	106.4	8.3
30	109.4	3.3	99.2	8.2	101.7	5.0	92.6	4.8

### 2.2. Content Determination of BMA in the Lyophilized Powder

The content of BMA in the lyophilized powder was determined as follows. Lyophilized powder was dissolved in pure water to obtain the decoction solution. The solution was vortexed for 3 min and then centrifuged at 13,000 rpm for 30 min. The supernatant was injected into the UPLC-MS/MS system for determination. The result showed that 0.92% of BMA was present in the lyophilized powder.

### 2.3. Pharmacokinetic Study

Although previous studies already investigated the pharmacokinetics of BMA [21,22], the present study is the first to compare the pharmacokinetic behavior of pure BMA and BMA as a marker compound of Wutou decoction in rats.

**Figure 5:**
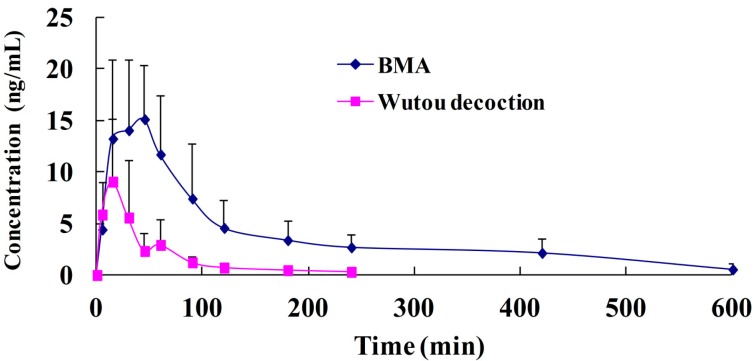
Plasma concentration* versus* time profile of BMA after oral administration of BMA and Wutou decoction at a dosage of 5 mg/kg (BMA). Each point represents the mean and standard deviation (mean ± SD) of five rats. BMA plasma concentrations at 240 min were approximately 0 after oral administration of Wutou decoction, and time points after that were deleted.

The developed UPLC-MS/MS method was used for the quantitation of plasma samples after oral administration of pure BMA (5 mg/kg) and Wutou decoction (0.54 g/kg, equivalent to BMA 5 mg/kg). As the predominant component of Wutou decoction, BMA exhibited the highest content in rat plasma after oral administration, which is in accordance with a previous study [21]. The plasma concentration* versus* time profile of BMA in the two rat groups is shown in Figure 5.

The pharmacokinetic parameters were expressed as the mean ± SD. All parameters are shown in Table 4. After the oral administration of pure BMA and Wutou decoction, the plasma concentration-time curve produced a fast rising trend followed by a sharp decline with *T*_1/2_ of 228.3 ± 117.0 min and 61.8 ± 35.1 min, respectively, until the levels fell below the detection limits. BMA was absorbed at a fast rate and reached a maximum concentration at C_max_ of 16.2 ± 6.7 ng/mL and 10.0 ± 5.8 ng/mL within 35.0 ± 11.2 min and 13.0 ± 4.5 min after pure BMA and Wutou decoction administration, respectively. The mean residence time (MRT) values for pure BMA were 155.0 ± 33.2 min, whereas that for Wutou decoction were 55.8 ± 16.4 min. The AUC_(0-t)_ values of BMA for pure BMA and Wutou decoction were 2247.4 ± 1171.9 and 447.8 ± 292.2 ng·min/mL, respectively, and the relative bioavailability of BMA after oral administration of Wutou decoction was 19.9%. Furthermore, the results of the pharmacokinetic study indicated that the absorption and elimination of BMA after the administration of pure BMA and Wutou decoction were very fast, within 13.0 min to 35.0 min and 61.8 min to 228.3 min, respectively.

**Table 4:** Pharmacokinetic parameters of BMA in Sprague-Dawley rat plasma (n = 5) after oral administration of BMA and Wutou decoction at 5 mg/kg (BMA). AUC_(0-t)_, *T*_1/2_, *C*_max_, *T*_max_, mean residence time (MRT) and relative bioavailability (RF) are shown in the table. Each value represents the mean and standard deviation (mean ± SD) of five rats.

Parameters	Unit	BMA	Wutou Decoction
AUC_(0-t)_	ng·min/mL	2,247.4 ± 1,171.9	447.8 ± 292.2 *
*T*_1/2_	min	228.3 ± 117.0	61.8 ± 35.1
*C*_max_	ng/mL	16.2 ± 6.7	10.0 ± 5.8
*T*_max_	min	35.0 ± 11.2	13.0 ± 4.5 *
MRT	min	155.0 ± 33.2	55.8 ± 16.4 *
RF	%	-	19.9

* *p* < 0.05 between two groups.

Notably, the pharmacokinetic process of BMA in Wutou decoction was significantly different compared with that in pure BMA (ANOVA, *p* < 0.05). AUC_(0-t)_, *T*_1/2_ and MRT decreased, suggesting that other complex ingredients in Wutou decoction might have an influence on the pharmacokinetics of BMA, which possibly demonstrated shorter retention time* in vivo* and lower distribution in the target organs, leading potentially to toxicity attenuation.

Numerous comparative pharmacokinetic studies have been reported on the absorption of effective components enhanced after combination with other herbs, some of which results from the competitive inhibition between components in herbs on the elimination via efflux transporters or the metabolism via drug metabolizing enzymes, such as CYP3A (microsomal cytochrome P450) [18]. The possible mechanism for the decrease in pharmacokinetic parameters is that the drug efﬂux transporters and CYP3A are distributed abundantly in the enterocytes of the gastrointestinal tract, which is the crucial place for the absorption of orally administered drugs. Therefore, the excretion by intestinal efflux transporters or metabolism by CYP3A might reduce drug absorption.

Although the exact mechanisms accounting for the different pharmacokinetic behaviors of BMA after pure BMA and Wutou decoction administration are not clear, different herb-herb interactions in Wutou decoction might be one possible explanation. The compounds in Wutou decoction might play an important role in affecting the elimination of BMA, influencing several links of ADME (absorption, distribution, metabolism and elimination) of BMA in rats, which leads to the decreased AUC and *T*_1/2_. For example, the efflux transporter, MRP2 (multi-drug resistance associate protein 2), was demonstrated to be involved in the efflux of BMA [6]. The diminished AUC_(0-t)_ might suggest that other complex ingredients in Wutou decoction reduced the absorption and bioavailability of BMA via induction of MRP2 efflux.

Furthermore, it was also reported that drug metabolizing enzymes, including CYP3A (microsomal cytochrome P450), played a significant role in the elimination of some herbs* in vitro* [23,24]. Meanwhile, BMA could be also metabolized by CYP3A [15]. Accelerating the metabolic elimination of BMA would reduce the oral bioavailability of BMA. Thus, another factor that may contribute to the faster elimination of BMA in Wutou decoction is metabolic induction by the other coexisting constituents. *T*_1/2_ and MRT decreased, which indicated that some components in Wutou decoction might induce the metabolism of CYP3A, thereby shortening the retention time of BMA* in vivo*, which might contributed to the toxicity reduction. Among all of the components of Wutou decoction, diammonium glycyrrhizinate reportedly has a positive effect on the toxicity reduction of aconitum alkaloids [25,26].

Thus, the faster elimination of BMA in rats after Wutou decoction administration is likely to be attributed to efflux through MRP2 or metabolism by CYP3A4/5 [6]. Some* in vivo* herb-herb interactions between the complex ingredients of Wutou decoction might exist to decrease the AUC and MRT of BMA via the enhanced expression of MRP2 or CYP3A. However, direct research on the induced expression of CYP3A or MRP2 by the complex ingredients in Wutou decoction is still limited, leading to more studies on a deeper level.

## 3. Experimental Section 

### 3.1. Chemicals and Reagents

BMA (≥98% purity) was obtained from the National Institute for the Control of Pharmaceutical and Biological Products (Beijing, China). Testosterone (≥98% purity) was purchased from Nacalai Tesque (Kyoto, Japan). Radix *Aconiti*, Herba *Ephedrae*, Radix *Paeoniae Alba*, Radix *Astragali* and Radix *Glycyrrhizae* (identified by the Sichuan Institute for Food and Drug Control) were purchased from Dongguan China Herbal Medicine Co., Ltd. (Dongguan, China). A voucher specimen was deposited at the Laboratory of Pharmaceutics, School of Pharmaceutical Sciences, Southern Medical University (Guangzhou, China). All other chemicals were of analytical grade.

### 3.2. Animals

Male Sprague-Dawley rats weighing between 230 and 280 g were supplied by the Laboratory Animal Center of Southern Medicine University (license: SCXK, Guangdong, 2006–0015) (Guangzhou, China). The rats were housed four per cage in a unidirectional airflow room under controlled temperature (20 °C to 24 °C), relative humidity (40% to 70%) and a 12-h light/dark cycle. The animal experimental protocol was approved by the ethics committee of Southern Medicine University (No: 2011–0015, Date: 24 October 2012). All animal studies were carried out according to the guide for care and use of laboratory animals. The rats were fasted, but allowed free access to water for at least 12 h before the experiment.

### 3.3. Instruments and Conditions

The UPLC-MS/MS system (Waters, Milford, MA, USA) consisted of a binary solvent manager, a column compartment, an auto-sampler manager and a single quadrupole mass spectrometer. All data acquisition was performed with Waters Masslynx V4.1 software.

The liquid chromatography separation of plasma samples was carried out on an Acquity UPLC BEH C18 column (1.7 μm; 2.1 mm × 50 mm) at 40 °C. Two micromolar ammonium acetate (A) and 100% acetonitrile (B) were used as the mobile phase. The flow rate was set at 0.3 mL/min. The gradients used for the elution were 0 min, 95% A; 0 min to 1.2 min, 95% to 88% A; 1.2 min to 3 min, 88% to 20% A; and 3 min to 4 min, 20% to 95% A. The injection volume was 5 μL.

Electro spray ionization in the positive mode was used to detect BMA in the plasma samples. The MS tune parameters were as follows: capillary voltage, 3.0 kV; cone voltage, 30 V; ion source temperature, 120 °C; desolvation temperature, 350 °C; desolvation gas flow, 650 L/h; and cone gas flow, 50 L/h. The mass spectrometer was operated in the multiple reaction monitoring (MRM) mode.

### 3.4. Preparation of Wutou Decoction Lyophilized Powder

Radix *Aconiti* (6 g) was added to 600 mL of boiling pure water and then boiled for 30 min to extract its active constituents. A mixture of *Herba Ephedrae* (9 g), Radix *Paeoniae Alba* (9 g), Radix *Astragali* (9 g) and Radix *Glycyrrhizae* (9 g) was added and boiled for 30 min. The solution was filtered and concentrated to obtain Wutou decoction. The decoction was concentrated to 100 mL using a rotary evaporator and then stored at −20 °C overnight for freezing. The frozen decoctions were freeze dried by a lyophilizer to obtain the lyophilized powder form of the decoction. The content of BMA in the lyophilized powder was quantified by UPLC-MS/MS the day before the pharmacokinetic experiment.

### 3.5. Preparation of Stock Solutions

A stock solution of BMA was prepared in methanol at a concentration of 5 mM, and the working solution of BMA was diluted with methanol. The testosterone stock solution was prepared in acetonitrile at a concentration of 20 mM, and the working solution of testosterone was diluted to 1000 nM with acetonitrile. All stock solutions were stored at −20 °C prior to use.

### 3.6. Biosample Collection

Pure BMA and Wutou decoction lyophilized powder was dissolved in 0.9% saline to obtain a concentration of 0.25 mg/mL (BMA). Ten rats were randomly divided into two groups. Pure BMA (5 mg/kg) and Wutou decoction (0.54 g/kg, equivalent to 5 mg/kg BMA) were orally administered to them, respectively. Serial blood samples (500 μL) were taken from the orbital sinus venous plexus at 0, 5, 15, 30, 45, 60, 90, 120, 180, 240, 420 and 600 min after gavage dosing. Each collected blood sample was centrifuged at 8000 rpm for 8 min. The plasma fractions were transferred into a disposable tube and then frozen at −80 °C until analysis.

### 3.7. Biosample Preparation

To detect BMA in the rat plasma, 80 μL of plasma was mixed with 320 μL methanol containing 100 nM testosterone. The mixture was centrifuged at 13,000 rpm for 30 min. A total of 300 μL of the supernatant was transferred to a disposable tube and then evaporated to dryness under a stream of nitrogen at room temperature. The residue was reconstituted with 100 μL of methanol-water (v:v = 1:1) and then injected into the UPLC-MS/MS system for analysis.

### 3.8. Method Validation

The specificity of the method was evaluated using blank plasma samples. Blank rat plasma was spiked with the working solutions of BMA (6 ng/mL) and testosterone (100 nM). The linearity of BMA in rat plasma was evaluated with the calibration curve based on the UPLC-MS/MS analysis of blank plasma spiked with different concentrations of BMA. This curve was obtained by plotting the peak area ratios* versus* the different concentrations of BMA. The LLOQ was defined based on the minimum concentration with a signal-to-noise ratio of ≥10. The precision and accuracy were evaluated by analyzing quality control (QC) samples with different concentrations. Intra-day and inter-day precision and accuracy were evaluated for 3 consecutive days with five replicates at each concentration per day.

The recoveries were determined by comparing the peak areas from the extracted samples with those from post-extracted blank plasma spiked with the analytes at the same concentration. The matrix effect was measured by comparing the peak areas of analytes added into the post-extracted blank with analytes dissolved in the matrix component-free reconstitution solvent. The stability of BMA in rat plasma was evaluated at room temperature for 12 h, after storage at −80 °C for 15 days, after three freeze-thaw cycles or in an auto-sampler for 12 h, respectively.

### 3.9. Data Analysis

The PK parameters were determined using the standard non-compartmental method and calculated using WinNonlin 5.2 (Pharsight, Mountain View, CA, USA). The *C*_max_ and the corresponding *T*_max_ were directly obtained from the raw data. The AUC to the last measurable concentration (AUC_(0-t)_) was calculated using the linear trapezoidal method. The relative bioavailability of BMA after oral administration of Wutou decoction was calculated as AUC_(decoction)_/AUC_(BMA)_ × 100%. An independent-sample *t*-test was performed twice to evaluate the differences of pharmacokinetic parameters between the two groups.

## 4. Conclusions

To the best of our knowledge, this study is the first to compare the pharmacokinetics of BMA after oral administration of pure BMA and Wutou decoction. The absorption of BMA in Wutou decoction was significantly reduced due to faster elimination, in comparison with that of pure BMA administration, which might contribute to toxicity reduction. Better understanding of the interactions between BMA and the coexisting ingredients in the Wutou decoction is still needed for clinical application of Wutou decoction.
